# Effect of μ-opioid agonist DAMGO on surface CXCR4 and HIV-1 replication in TF-1 human bone marrow progenitor cells

**DOI:** 10.1186/1756-0500-7-752

**Published:** 2014-10-23

**Authors:** Marianne Strazza, Anupam Banerjee, Aikaterini Alexaki, Shendra R Passic, Olimpia Meucci, Vanessa Pirrone, Brian Wigdahl, Michael R Nonnemacher

**Affiliations:** Department of Microbiology and Immunology, Drexel University College of Medicine, 245 N. 15th Street, MS# 1013A, Philadelphia, PA 19102 USA; Center for Molecular Virology and Translational Neuroscience, Drexel University College of Medicine, Philadelphia, PA 19102 USA; Center for Neuroimmunology and CNS Therapeutics, Institute for Molecular Medicine and Infectious Disease, Drexel University College of Medicine, Philadelphia, PA 19102 USA; Department of Pharmacology and Physiology, Drexel University College of Medicine, Philadelphia, PA 19102 USA

**Keywords:** μ-opioid receptor (MOR-1), DAMGO, Human immunodeficiency virus type 1 (HIV-1), Bone marrow, CXCR4

## Abstract

**Background:**

Approximately one-third of the AIDS cases in the United States have been attributed to the use of injected drugs, frequently involving the abuse of opioids. Consequently, it is critical to address whether opioid use directly contributes to altered susceptibility to HIV-1 beyond the increased risk of exposure. Previous in vitro and in vivo studies addressing the role of μ-opioid agonists in altering levels of the co-receptor CXCR4 and subsequent HIV-1 replication have yielded contrasting results. The bone marrow is believed to be a potential anatomical sanctuary for HIV-1.

**Methods:**

The well-characterized CD34^+^CD38^+^ human bone marrow–derived hematopoietic progenitor cell line TF-1 was used as a model to investigate the effects of the μ-opioid receptor–specific peptide DAMGO (D-Ala2,N-Me-Phe4, Gly5-ol-enkephalin) on CXCR4 expression as well as infection of undifferentiated human hematopoietic progenitor cells.

**Results:**

The results revealed the presence of the μ-opioid receptor-1 isoform (MOR-1) on the surface of TF-1 cells. Furthermore, immunostaining revealed that the majority of TF-1 cells co-express MOR-1 and CXCR4, and a subpopulation of these double-positive cells express the two receptors in overlapping membrane domains. Three subpopulations of TF-1 cells were categorized based on their levels of surface CXCR4 expression, defined as non-, low-, and high-expressing. Flow cytometry indicated that treatment with DAMGO resulted in a shift in the relative proportion of CXCR4^+^ cells to the low-expressing phenotype. This result correlated with a >3-fold reduction in replication of the X4 HIV-1 strain IIIB, indicating a role for the CXCR4 high-expression subpopulation in sustaining infection within this progenitor cell line.

**Conclusions:**

These experiments provide insight into the impact of μ-opioid exposure with respect to inhibition of viral replication in this human TF-1 bone marrow progenitor cell line model.

## Background

In addition to several studies linking chronic opioid use to immunomodulation [1] and increased susceptibility to bacterial infections [2], the role of opiates as potential cofactors in HIV-1 pathogenesis and disease has also been proposed. In vitro experiments that involve treatment of peripheral blood mononuclear cells with morphine prior to HIV-1 exposure resulted in increased viral replication [3]. It is now known that prolonged treatment with morphine or the selective μ-opioid receptor agonist D-Ala2,N-Me-Phe4,Gly5-ol-enkephalin (DAMGO) enhances the percentage of T cells and monocytes expressing the HIV-1 co-receptors CXCR4 and CCR5, respectively, thereby increasing the number of infected cells and the overall amount of infectious virus produced in subsequent experiments [4]. More directly, morphine treatment increases HIV-1 infection of blood monocyte–derived macrophages by upregulating CCR5 expression and inhibiting production of β-chemokines, endogenous CCR5 ligands [5].

Ongoing in vivo studies performed in the simian immunodeficiency virus (SIV)-infected rhesus macaque/model have yielded a better understanding of the impact of prolonged morphine exposure on HIV-1 pathogenesis. Prolonged morphine exposure increased viral replication [6, 7], increased the number of SIV-infected T cells [8], accelerated disease progression and neuropathogenesis [7], increased the amount of plasma virus [6, 7], and increased the incidence of mortality [7]. Despite these numerous studies, a direct link between an alteration in CXCR4 or CCR5 surface expression levels and quantity of plasma virus has not been established.

The μ-opioid receptor-1 isoform (MOR-1), the best characterized isoform of the μ-opioid receptor family, has been found on cellular subsets of the immune system, as well as cells of the central nervous system, including but not limited to neurons [9–11]. It is possible that reported inconsistencies in the literature regarding the expression profile of CXCR4 may be attributable to a cell type–specific regulation of this chemokine co-receptor by μ-opioids. This process in turn might translate into the differential ability of μ-opioids to modulate HIV-1 replication in divergent cellular populations. To investigate the effect of μ-opioids on CXCR4 expression in human bone marrow progenitor cells, the TF-1 cell line was used; it represents a model of susceptible CD34^+^/CD38^+^ human hematopoietic progenitor cells that are blocked at an early stage of differentiation [12]. To begin experimentation in the TF-1 cell line, experiments were performed to assess levels of MOR-1 in these cells by western immunoblot analyses, flow cytometry, and immunofluorescence microscopy. To analyze the relative surface distribution of MOR-1 and CXCR4, immunofluorescence microscopy studies were also performed. Alterations in total CXCR4 protein levels in DAMGO-treated TF-1 cells were determined using western immunoblot analyses and surface expression levels were examined using flow cytometry. We have previously demonstrated that, in addition to CXCR4, TF-1 cells express the primary HIV-1 receptor CD4 on their cell surface, thereby supporting productive infection by the HIV-1 X4-utilizing (X4) IIIB strain [13]. This observation prompted studies examining the consequence of DAMGO-mediated perturbation in CXCR4 levels on HIV-1 X4 replication in this human bone marrow–derived progenitor cell population.

## Results

### Identification of MOR-1 in TF-1 cells

**Figure 1:**
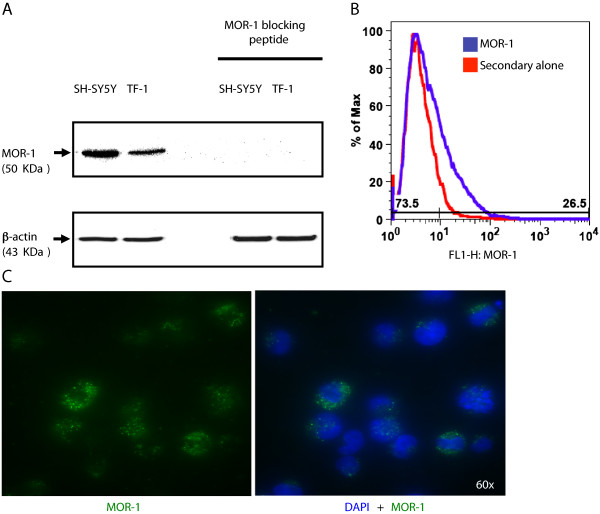
**MOR-1 is expressed on the surface of TF-1 cells. (A)** Western immunoblot analyses performed with the human neuroblastoma cell line SH-SY5Y (positive control) and TF-1 cell line whole cell lysates demonstrate the presence of μ-opioid receptor-1 protein (MOR-1) at ~50 KDa. Abrogation of the 50-KDa band with preincubation of the polyclonal antibody with a MOR-1 blocking peptide confirms the specificity of reaction. The membrane was stripped and reblotted for human β-actin (43 KDa) as the endogenous loading control. The blot is representative of results obtained from three independent experiments. **(B)** Flow cytometry of nonpermeabilized TF-1 cells stained with an antibody directed against a region mapping within the N-terminus of MOR-1 (20 μg/ml) shows expression on the cell surface. A fluorescein isothiocyanate (FITC)-conjugated secondary antibody directed against the primary host was used in combination with primary staining (blue) or alone (red) as a negative control. **(C)** The left panel shows a subpopulation of paraformaldehyde-fixed, nonpermeabilized TF-1 cells expressing MOR-1 on the surface (detected in green with a FITC-tagged antibody directed against a region mapping within the N-terminus of MOR-1). The right panel shows a merge with DAPI (4',6-diamidino-2-phenylindole) nuclear staining of the same field. The images were taken at 60× with an Olympus IX81 deconvolution microscope.

Western immunoblot analysis confirmed the presence of MOR-1 protein within TF-1 cells, clearly demonstrating the existence of a specific protein species at approximately 50 KDa, the expected molecular mass of human MOR-1 (Figure 1A) [14]. As expected, the levels of MOR-1 in undifferentiated SH-SY5Y neuroblastoma cell lysates (positive control) were much higher than those observed in TF-1 lysates. In addition, the detection of MOR-1 was abrogated by preincubating the primary antibody with the MOR-1 blocking peptide. Blotting the membrane for β-actin confirmed equal loading in all lanes. Immunostaining of nonpermeabilized cells analyzed by flow cytometry (Figure 1B) and fluorescent microscopy (Figure 1C) clearly demonstrated that MOR-1 was expressed at the cell surface.

### Relative localization of MOR-1 and CXCR4

**Figure 2:**
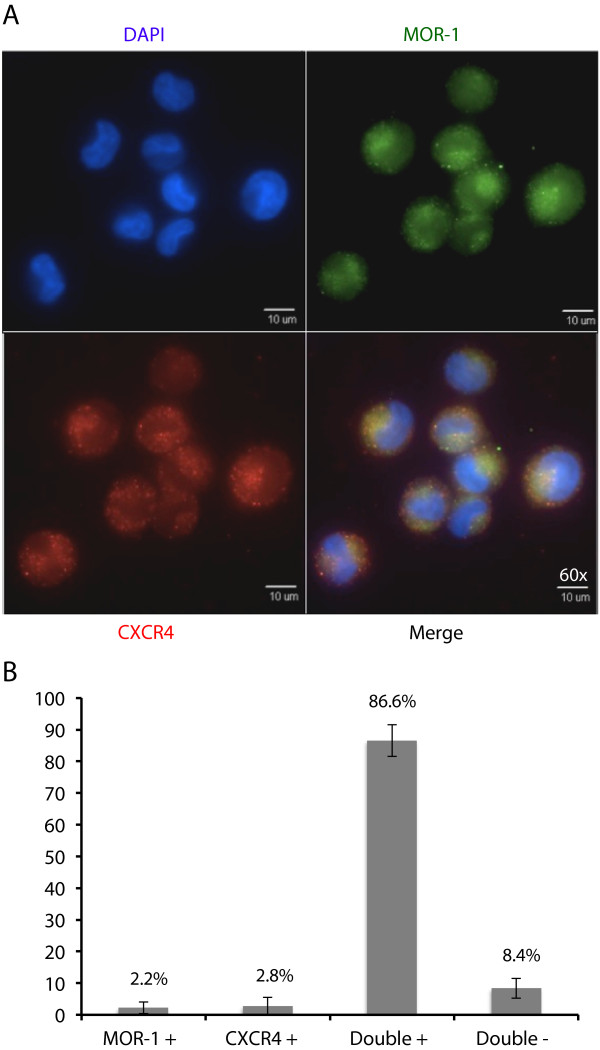
**TF-1 cells express MOR-1 and CXCR4 in overlapping domains. (A)** TF-1 cells were cultured and stained as described above. Briefly, TF-1 cells were grown on poly-D-lysine coated coverslips and stained, nonpermeabilized, with primary antibodies directed against MOR-1 and CXCR4, and with DAPI (4',6-diamidino-2-phenylindole) staining to show nuclear morphology. The upper left panel demonstrates a nuclear reaction with DAPI (in blue). The upper right panel demonstrates a surface reaction pattern for MOR-1 (in green). The bottom left panel also demonstrates a surface reaction pattern for CXCR4 (in red). The bottom right panel is an overlay showing the relative expression of MOR-1 and CXCR4. These images are representative of all fields captured. All images were obtained at 60× with an Olympus IX81 deconvolution microscope. **(B)** TF-1 cells were quantified by counting a total of 1000 cell nuclei, and these cells were then characterized based on MOR-1 and CXCR4 staining as single-positive for either protein, double-positive, or double-negative. This analysis shows that the majority of TF-1 cells are double-positive for surface expression of the two proteins.

To analyze the relative distribution of MOR-1 and CXCR4 at the cellular level, immunofluorescence studies on nonpermeabilized TF-1 cells was performed. CXCR4 and MOR-1 were shown to be co-expressed on the surface of a number of TF-1 cells. The majority of the cells quantitated (87%) expressed both MOR-1 and CXCR4 at the cell surface (Figure 2). Far fewer cells expressed MOR-1 or CXCR4 exclusively (2% and 3% of counted cells, respectively), and 8% of cells quantitated did not express either receptor.

**Figure 3:**
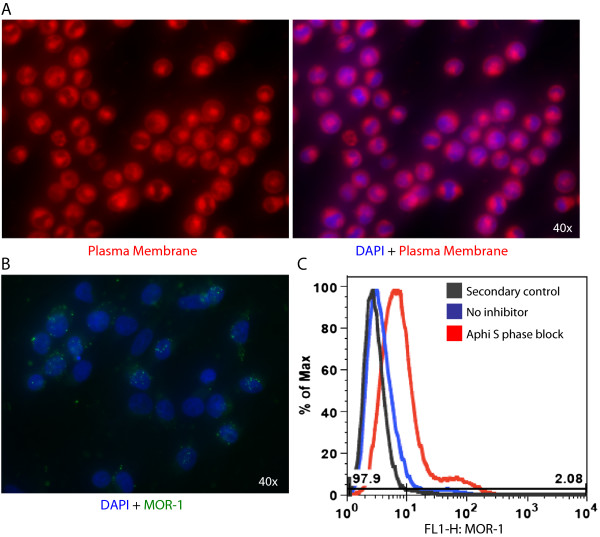
**MOR-1 surface localization varies with the phase of the cell cycle. (A)** TF-1 cells were grown on poly-D-lysine coverslips and left nonpermeabilized. Cells were then stained with a fluorescently tagged amphipathic molecule that embeds within the lipid bilayer. The molecule embeds preferentially into regions of high lipid density, and therefore these regions are represented by high fluorescence. **(B)** TF-1 cells were grown on poly-D-lysine coverslips and then treated with aphidicolin (10 μg/ml) for 16 hours to induce arrest in S phase. Following treatment, cells were left nonpermeabilized and stained for MOR-1 and with DAPI (4',6-diamidino-2-phenylindole). MOR-1 puncta are located diffusely throughout the cell. All images were obtained at 40× with an Olympus IX81 deconvolution microscope. **(C)** TF-1 cells were left untreated or arrested in S phase and stained for MOR-1 as described above, then analyzed by flow cytometry. Analysis shows an increase in MOR-1 expression in S phase, as indicated by an increase in mean fluorescent intensity.

Interestingly, on the surface of double-positive cells, the staining for the two receptors appears to overlap, suggesting the possibility of physical proximity. A proportion of these double-positive cells co-expressed CXCR4 and MOR-1 in distinct domains on the surface that traced the circumference of the nucleus. Additionally, double-positive cells that exhibited uniform distribution of expression of the two receptors across the cell surface differed in nuclei morphology. A similar co-localization pattern of MOR-1 and CXCR4 has been previously reported in cortical neurons [10]. Given the observed changes in nuclear morphology, we proceeded to determine whether these discrete domains were specific to MOR-1 and CXCR4 or resulted from general TF-1 cellular topography. To distinguish between these two possibilities, fluorescently tagged amphipathic molecules that embed within regions of high lipid density were used to visualize the plasma membrane. This procedure clearly demonstrated that the observed discrete domains were regions of high lipid density (Figure 3A), likely owing to membrane thinning and nuclear dispersion during M phase of the cell cycle.

Together these observations suggest a possible link between cell cycle and the observed MOR-1 and CXCR4 surface localization patterns. To better understand the influence of the phase of the cell cycle on MOR-1 and CXCR4 cell surface localization, TF-1 cells were treated with aphidicolin (10 μg/ml) for 16 hours to induce an arrest in early S phase. Following treatment, MOR-1 expression was analyzed on the surface of TF-1 cells in S phase using fluorescent microscopy and flow cytometry. TF-1 cells in S phase expressed MOR-1 in a punctate pattern across the cell surface (Figure 3B) and expressed MOR-1 at a higher mean fluorescent intensity (MFI) than the untreated, heterogeneous population of TF-1 cells (Figure 3C). These observations suggested that the diverse cell surface expression patterns of MOR-1 in conjunction with CXCR4 are the result of a specific phase of the cell cycle. While the two receptors are expressed in overlapping membrane domains, suggesting the possibility of proximity, the localization in discrete membrane domains surrounding the nucleus may not be due to regulated trafficking of the two receptors.

### Effects of prolonged DAMGO exposure on CXCR4 expression

**Figure 4:**
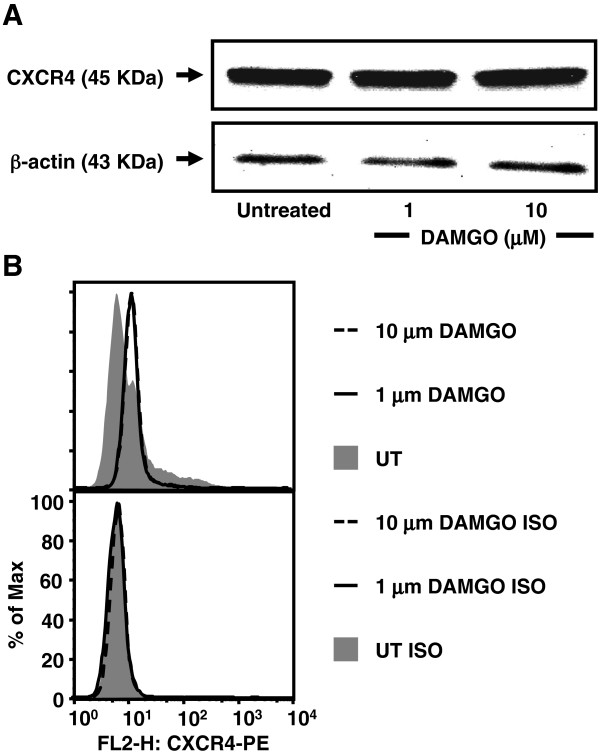
**DAMGO alters surface expression of CXCR4 on TF-1 cells. (A)** Total levels of CXCR4 remain unaltered in TF-1 cells upon DAMGO treatment. Western immunoblot analyses performed with TF-1 cell line whole cell lysates demonstrate the presence of glycosylated CXCR4 monomer at 45 KDa. In comparison to the untreated cells, DAMGO (1 and 10 μM)-treated cells exhibited a similar density of CXCR4, indicating that CXCR4 expression at the protein level does not change. The membrane was stripped and reblotted for human β-actin (43 KDa) as the endogenous loading control. **(B)** Treatment of TF-1 cells with DAMGO results in a shift in CXCR4^+^ cells to the low-expressing phenotype. Immunofluorescence flow cytometry analysis of nonpermeabilized TF-1 cells was performed to detect a change in surface expression of CXCR4 following DAMGO treatment. Immunofluorescence analysis for surface CXCR4 by flow cytometry demonstrated that TF-1 cells were distributed among three populations, non-expressing cells, low-expressing cells (lower mean fluorescence intensity [MFI]), and high-expressing cells (higher MFI). DAMGO pretreatment (1 and 10 μM) for 24 hours resulted in a shift in the relative proportion of CXCR4^+^ cells to the low-expressing phenotype, as is evident from the increase in the lower MFI peak and the reduction in the higher MFI peak. CXCR4 antibody isotype controls are shown at the bottom.

To study the effects of MOR-1 signaling on CXCR4 expression, the levels of total as well as surface CXCR4 was examined in DAMGO-pretreated TF-1 cells. Western immunoblot analyses were performed on whole cell lysates prepared from TF-1 cells, in the absence or presence of two different concentrations of DAMGO (1 and 10 μM), a specific MOR-1 agonist. Total levels of CXCR4 protein (glycosylated CXCR4 monomer detected at 45 KDa) remained unchanged, suggesting that DAMGO treatment does not affect overall expression or stability of the CXCR4 protein within the cell (Figure 4A). This observation prompted studies to determine whether the subcellular localization of CXCR4 was affected by DAMGO treatment. Immunostaining for surface CXCR4 was performed on nonpermeabilized TF-1 cells and analyzed by flow cytometry. Untreated TF-1 cells displayed a distribution among a range of MFIs, with three distinct cellular populations: a large population of CXCR4 non-expressing cells, a low-expressing cell population, and a smaller population of CXCR4 high-expressing cells (Figure 4B). This is consistent with the heterogeneity in CXCR4 expression observed in the human hematopoietic stem cell compartment [15] and is consistent with levels of CXCR4 surface expression in primary human bone marrow cells. DAMGO (1 and 10 μM) pretreatment for 24 hours resulted in an alteration in the relative proportion of CXCR4^+^ cells toward the low-expressing phenotype, which was evident from an increase in the low-expressing (midrange) MFI peak and a reduction in both the non-expressing and high-expressing MFI peaks. Collectively, these studies show that total protein levels of CXCR4 remain unchanged, while surface expression levels of CXCR4 are altered following DAMGO exposure.

### Effects of prolonged DAMGO exposure on HIV-1 replication in TF-1 cells

**Figure 5:**
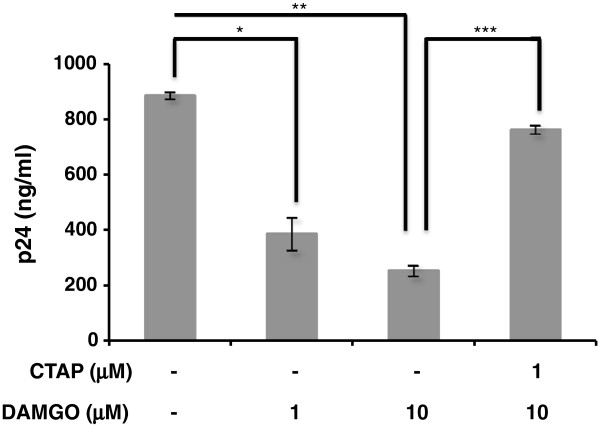
**DAMGO treatment inhibits the replication of HIV-1 X4-utilizing strain IIIB in TF-1 cells.** Analyses involving the detection of HIV-1 p24 capsid protein were performed on cellular supernatants 24 hours after infection to assess HIV-1 IIIB replication in response to DAMGO treatment of TF-1 cells. Absolute values of p24 levels are denoted in ng/ml on the Y axis. A 2.3-fold decrease was observed with DAMGO treatment (1 μM), which further declined to 3.5-fold at 10 μM. CTAP pretreatment reversed DAMGO-mediated decline in p24 levels. Samples were assayed in triplicate and results shown are the average of two independent experiments. *p = 0.00012, **p =  0.00000076, and *** p = 0.000002.

Given the observed changes in the level of CXCR4 surface expression following DAMGO treatment and the understanding that co-receptor expression levels correlate with cell susceptibility to HIV-1 infection [16], the level of impact DAMGO would have on HIV-1 replication was of interest. HIV-1 core antigen (p24) assays were used to assess HIV-1 replication in TF-1 cells in the presence of DAMGO. In comparison with untreated cells, DAMGO pretreatment for 24 hours resulted in a dose-dependent decline in p24 production (Figure 5). Specifically, p24 levels dropped from 885 ng/ml in untreated cells to 386 ng/ml in cells treated with DAMGO at 1 μM (p < 0.001) and 254 ng/ml in cells treated with DAMGO at 10 μM (p < 0.001). This decline, which ranged from 2.3- to 3.5-fold, was partially reversed upon CTAP (D-Phe-Cys-Tyr-D-Trp-Arg-Thr-Pen-Thr-NH) pretreatment to 763 ng/ml (p < 0.001). Altogether, the results suggested that exposure to μ-opioids might render bone marrow progenitor cell populations less susceptible to HIV-1 infection.

## Discussion

Because HIV-1 has been reported to penetrate the bone marrow [17, 18] of infected patients, it has been suggested that cellular subsets of this important compartment may serve as reservoirs for the virus. We aimed to determine the effects of the specific MOR-1 agonist DAMGO on viral replication in human bone marrow progenitor cells. The human CD34^+^/CD38^+^ TF-1 bone marrow–derived hematopoietic progenitor cell line was used as an in vitro model because it is known to express the HIV-1 receptor CD4 as well as the co-receptor CXCR4.

MOR-1 is the most extensively characterized receptor subtype of the family and it has been identified on terminally differentiated mature immune cell populations, including human CD4^+^ cells, cells of the monocyte-macrophage lineage [19], and more primitive CD34^+^38^−^ peripheral blood and cord blood stem cells [20]. Its presence on more terminally differentiated progenitor cells and the concomitant underlying significance of its presence have, however, not been fully elucidated. In this regard, we have clearly demonstrated the presence of MOR-1 in a TF-1 human bone marrow progenitor cell population.

MOR-1 has also been shown to oligomerize with other G protein–coupled receptors (GPCRs) such as the chemokine co-receptor CCR5 [21] with an adverse impact observed on the G-protein coupling of the other oligomerization partner. Alterations in G-protein coupling, differential affinity of specific agonists, and enhanced internalization have also been reported for the μ-δ opioid receptor (MOR-DOR) hetero-oligomeric complex [22]. Therefore, the presence of MOR-1 and CXCR4 in overlapping domains on TF-1 cells leaves open the possibility of dimerization between these receptors. Such localization of MOR-1 has also been reported in neuronal membranes of the rat caudate putamen wherein μ-opioid receptors were found in cholesterol and sphingolipid-rich membrane subdomains known as lipid rafts [20], where they seem to be important in regulation of GPCR signaling, receptor phosphorylation, and membrane trafficking [23]. This observation raises interesting questions concerning the biological significance of MOR-1 localization in TF-1 progenitor cells. The results reported herein have also revealed MOR-1-mediated alteration in surface CXCR4 levels, in line with our previous studies [10, 11]. It has been previously reported that CXCR4 signaling is impeded by DAMGO in rat cortical neurons [10, 11]. Therefore, future studies will be focused on investigating whether molecular events triggered by MOR-1 signaling affect CXCR4 receptor recycling and/or CXCR4 signaling in bone marrow progenitor cells.

These studies also provide a unique insight into the role of μ-opioids in modulating HIV-1 replication in bone marrow progenitor cells. In the setting of HIV-1 infection, downregulation of CXCR4 might translate into a reduction in HIV-1 replication under the influence of μ-opioids, thereby serving a protective function, as suggested in studies performed in macaques [24]. As shown here, DAMGO exposure resulted in a decrease in HIV-1 replication. While this decrease may be the result of a decrease in viral entry involving CXCR4, the possibility remains that this decrease is the result of inhibition of post-entry events in the viral life cycle. Paradoxically, because the SDF-1–CXCR4 axis is vital for survival of progenitor cells [25], a reduction in levels of CXCR4 might render them more susceptible to apoptosis in vivo. This result might provide a plausible explanation for the hematopoietic dysregulation observed in animal models of long-term morphine administration [26–29]. These results warrant further studies aimed at dissecting the divergent and dichotomous effects of μ-opioids on terminally differentiated cell populations in the peripheral blood and bone marrow.

## Conclusions

These studies have demonstrated the presence of both MOR-1 and CXCR4 on the cell surface of the hematopoietic progenitor cell line TF-1. Additionally, these receptors are localized to regions of high hydrophobicity within the membrane, indicative of high lipid density. As such, MOR-1 and CXCR4 are localized in proximity in this cell population. Most importantly, these experiments demonstrate the alteration in surface expression of CXCR4 as a result of DAMGO stimulation, and reduction in HIV-1 replication of a CXCR4-utilizing (X4) viral strain.

## Methods

### Materials

The potent cAMP phosphodiesterase inhibitor 3-isobutyl-1-methylxanthine was obtained from Sigma-Aldrich (St. Louis, MO). DAMGO, a selective μ-opioid peptide, and D-Phe-Cys-Tyr-D-Trp-Arg-Thr-Pen-Thr-NH2 (CTAP), a selective MOR antagonist, were purchased from Sigma-Aldrich. Amicon Ultra-0.5, Ultracel-3 Membrane, 3-kDa centrifugal filters for protein purification were obtained from Millipore (Bellerica, MA). Whole cell lysates were prepared for protein studies in radioimmunoprecipitation assay (RIPA) buffer (Pierce ECL; Thermo Fisher Scientific, Rockford, IL), and protein concentrations were calculated using the bicinchoninic acid (BCA) protein assay as described by the manufacturer (Pierce ECL).

### Cell culture and treatment procedures

The TF-1 CD34^+^38^+^ cell line (American Type Culture Collection [ATCC], Manassas, VA) was grown in TF-1 media which is composed of RPMI-1640 medium (ATCC; Mediatech, Inc., Manassas, VA) supplemented with 10% heat-inactivated fetal bovine serum (FBS) (HyClone, Thermo Fisher Scientific, Logan, UT), penicillin (100 U/ml), streptomycin (100 μg/ml), and recombinant human granulocyte/macrophage colony-stimulating factor (2 ng/ml) (eBioscience, San Diego, CA). The SH-SY5Y human neuroblastoma cell line (ATCC) was propagated in a 1:1 mixture of Eagle’s minimal essential medium with Earle’s salts, L-glutamine, and F-12 K nutrient mixture (Mediatech) supplemented with 10% heat-inactivated FBS (HyClone), penicillin (100 U/ml), and streptomycin (100 μg/ml). The cells were maintained at 37°C in 5% CO_2_ at 90% relative humidity.

### Western immunoblot analysis

To identify MOR-1 at the protein level, an N-terminus-specific antibody was used that recognizes a region mapping within the extracellular domain of human MOR-1 (N-20, Santa Cruz Biotechnology, Santa Cruz, CA). To demonstrate the specificity of interaction, assays were performed under nonblocking and blocking conditions. Whole cell lysates were prepared in RIPA buffer (Pierce ECL) and used to determine protein concentrations utilizing the BCA protein assay as described by the manufacturer (Pierce ECL). An equal amount of protein was run on a 10% sodium dodecyl sulfate–polyacrylamide gel and transferred to a 0.45-μm Immobilon-P polyvinylidene fluoride membrane followed by probing with the MOR-1 polyclonal antibody and detecting with a horseradish peroxidase (HRP)-conjugated antigoat IgG antibody (Jackson ImmunoResearch Laboratories, West Grove, PA). As a negative control, the primary antibody was preincubated with a MOR-1-specific blocking peptide (1:1) (Santa Cruz Biotechnology) before probing for the receptor protein. Because SH-SY5Y neuroblastoma cells are known to constitutively express high levels of MOR-1 [30], they were used as a positive control in the experiments. Equal loading was confirmed by stripping the membrane using the Restore Western Blot Stripping Buffer (Thermo Scientific) and reblotting for β-actin with a mouse monoclonal antibody from Sigma-Aldrich. To study the total CXCR4 protein expression resulting from prolonged DAMGO exposure, TF-1 cells were first starved for 1 hour. After washing in 1× phosphate-buffered saline (PBS), aliquots were kept untreated or treated with DAMGO (1 or 10 μM) for 24 hours followed by lysis and processing for western immunoblot analysis using a polyclonal anti-CXCR4 antibody (H-118) (Santa Cruz Biotechnology) and an HRP-conjugated antirabbit IgG antibody (Jackson ImmunoResearch Laboratories). All immunoblots were visualized using a chemiluminescent detection procedure as described by the manufacturer (Pierce ECL) on a ChemiDoc acquisition/analysis station (Bio-Rad Laboratories, Hercules, CA).

### Immunofluorescence analysis

To examine cellular distribution of MOR-1 and to compare the expression pattern of MOR-1 with that of CXCR4 on the surface of TF-1 cells, immunofluorescence studies were performed. Cells were incubated on poly-D-lysine-coated coverslips. Where indicated, cells were treated for 16 hours with aphidicolin (10 μg/ml) to arrest cells in early S phase. Cells were subsequently fixed with 2% followed by 4% paraformaldehyde. So that we could visualize the surface expression of the two receptors, TF-1 cells were not permeabilized prior to staining. Sequential staining began with primary labeling with an N-terminus-specific MOR-1 antibody, N-20 (Santa Cruz Biotechnology), followed by secondary labeling with a fluorescein isothiocyanate-conjugated antigoat IgG antibody. Subsequently, CXCR4 was labeled with an N-terminus-specific phycoerythrin-conjugated antihuman CXCR4 monoclonal antibody, 1D9 (Pharmingen, BD Biosciences, San Jose, CA), followed by secondary staining with a rhodamine (TRITC)-conjugated antirat IgG antibody (Jackson ImmunoResearch Laboratories). Each step was accompanied by washing three times with PBS. Negative controls (not shown) consisted of “no primary” and “isotype primary” antibody conditions. Cells were mounted with Vectashield mounting medium (Vector Laboratories, Burlingame, CA) and observed under an Olympus IX81 deconvolution microscope at 60× magnification. A total of 1000 cells were counted following three independent staining experiments, and the percentage of cells in each phenotype was calculated based on the total number of cells counted. Averages reported were calculated based on the three independent experiments, and error bars represent the standard deviation of the results.

### Flow cytometry

To determine mean fluorescence intensity of surface CXCR4 on TF-1 cells, the cells were serum starved for 1 hour, then resuspended in TF-1 serum free media. Aliquots of 5 × 10^5^ cells were incubated in the absence or presence of two different concentrations of DAMGO (1 and 10 μM) for 24 hours. These concentrations were selected because 10 μM represents the upper range of morphine observed in the serum of morphine-dependent animals [31]. With respect to the MOR-1 analysis, where indicated, cells were incubated in the absence or presence of aphidicolin (10 μg/ml) for 16 hours to arrest cells in early S phase. Collected cells were then washed with FACS wash buffer (Hanks balanced saline solution [Mediatech], 3% FBS, and 0.02% NaN3). So that we could visualize the surface expression of the two receptors, TF-1 cells were not permeabilized prior to staining. Cells were then reacted with a titrated amount of a monoclonal phycoerythrin-conjugated antihuman CXCR4 antibody 12G5 (R & D Systems, Minneapolis, MN) or MOR-1 (N-20, Santa Cruz Biotechnology) on ice for 30 minutes. Concentration-matched IgG2A isotype or whole IgG control antibody preparations were used as negative controls. Subsequently, cells were again washed with FACS wash buffer and fixed with 1% paraformaldehyde. We performed flow cytometry using a FACSCalibur Flow Cytometer (BD Biosciences) and analyzed the results using FlowJo version 6.1.1 software (Tree Star, Ashland, OR).

### HIV-1 p24 ELISA

To assess the effects of DAMGO pretreatment on HIV-1 replication in TF-1 cells, p24 assays for measuring viral core antigen were performed. Cells were seeded into 12-well plates at a density of 0.5 × 10^6^ cells/ml and pretreated for 24 hours with DAMGO (1 or 10 μM) alone or DAMGO (10 μM) and CTAP (1 μM) in TF-1 serum free media. After 24 hours, treated cells were seeded in a new 12-well plate at a concentration of 0.5 × 10^6^ cells/ml/well. This step was followed by exposure to the X4-utilizing (X4) HIV-1 IIIB strain at a titer of 10^5^ median tissue culture infective dose (TCID_50_) (Advanced Biotechnologies, Inc., Columbia, MD). Two hours after infection, cells were washed with PBS, resuspended in TF-1 media containing serum, and reseeded in 12-well plates. Following incubation for 24 hours, the supernatant was collected and assayed for p24 core antigen using an Alliance p24 ELISA procedure as described by the manufacturer (Perkin Elmer, Waltham, MA). All samples were assayed in triplicate and p24 values were normalized for 1 × 10^6^ cells/ml.

### Statistical analysis

Significance was determined using the student *t*-test, considering p values of <0.05 significant.

## Abbreviations

BCA: Bicinchoninic acid
CTAP: D-Phe-Cys-Tyr-D-Trp-Arg-Thr-Pen-Thr-NH
DAMGO: D-Ala2,N-Me-Phe4,Gly5-ol-enkephalin
DAPI: 4′,6-diamidino-2-phenylindole
FBS: Fetal bovine serum
FITC: Fluorescein isothiocyanate
HRP: Horseradish peroxidase
MFI: Mean fluorescent intensity
MOR-1: μ-opioid receptor-1
PBS: Phosphate-buffered saline
SIV: Simian immunodeficiency virus.
